# mTORC1 and mTORC2 Complexes Regulate the Untargeted Metabolomics and Amino Acid Metabolites Profile through Mitochondrial Bioenergetic Functions in Pancreatic Beta Cells

**DOI:** 10.3390/nu14153022

**Published:** 2022-07-22

**Authors:** Ghada A. Soliman, Rinat R. Abzalimov, Ye He

**Affiliations:** 1 Department of Environmental, Occupational, and Geospatial Health Sciences, Graduate School of Public Health and Health Policy, City University of New York, New York, NY 10027, USA; 2 Structural Biology Initiative, Advanced Science Research Center (ASRC), City University of New York, New York, NY 10031, USA; rabzalimov@gc.cuny.edu; 3 Neuroscience Initiative, Advanced Science Research Center (ASRC), City University of New York, 85 St. Nicholas Terrace, New York, NY 10031, USA; yhe1@gc.cuny.edu

**Keywords:** mTORC1, mTORC2, high-resolution mass spectrometry (HRMS), mitochondrial stress, oxygen consumption rate (OCR), extra cellular acidification rate (ECAR), the internal exposome

## Abstract

Background: Pancreatic beta cells regulate bioenergetics efficiency and secret insulin in response to glucose and nutrient availability. The mechanistic Target of Rapamycin (mTOR) network orchestrates pancreatic progenitor cell growth and metabolism by nucleating two complexes, mTORC1 and mTORC2. Objective: To determine the impact of mTORC1/mTORC2 inhibition on amino acid metabolism in mouse pancreatic beta cells (Beta-TC-6 cells, ATCC-CRL-11506) using high-resolution metabolomics (HRM) and live-mitochondrial functions. Methods: Pancreatic beta TC-6 cells were incubated for 24 h with either: RapaLink-1 (RL); Torin-2 (T); rapamycin (R); metformin (M); a combination of RapaLink-1 and metformin (RLM); Torin-2 and metformin (TM); compared to the control. We applied high-resolution mass spectrometry (HRMS) LC-MS/MS untargeted metabolomics to compare the twenty natural amino acid profiles to the control. In addition, we quantified the bioenergetics dynamics and cellular metabolism by live-cell imaging and the MitoStress Test XF24 (Agilent, Seahorse). The real-time, live-cell approach simultaneously measures the oxygen consumption rate (OCR) and extracellular acidification rate (ECAR) to determine cellular respiration and metabolism. Statistical significance was assessed using ANOVA on Ranks and post-hoc Welch *t*-Tests. Results: RapaLink-1, Torin-2, and rapamycin decreased L-aspartate levels compared to the control (*p* = 0.006). Metformin alone did not affect L-aspartate levels. However, L-asparagine levels decreased with all treatment groups compared to the control (*p* = 0.03). On the contrary, L-glutamate and glycine levels were reduced only by mTORC1/mTORC2 inhibitors RapaLink-1 and Torin-2, but not by rapamycin or metformin. The metabolic activity network model predicted that L-aspartate and AMP interact within the same activity network. Live-cell bioenergetics revealed that ATP production was significantly reduced in RapaLink-1 (122.23 ± 33.19), Torin-2 (72.37 ± 17.33) treated cells, compared to rapamycin (250.45 ± 9.41) and the vehicle control (274.23 ± 38.17), *p* < 0.01. However, non-mitochondrial oxygen consumption was not statistically different between RapaLink-1 (67.17 ± 3.52), Torin-2 (55.93 ± 8.76), or rapamycin (80.01 ± 4.36, *p* = 0.006). Conclusions: Dual mTORC1/mTORC2 inhibition by RapaLink-1 and Torin-2 differentially altered the amino acid profile and decreased mitochondrial respiration compared to rapamycin treatment which only blocks the FRB domain on mTOR. Third-generation mTOR inhibitors may alter the mitochondrial dynamics and reveal a bioenergetics profile that could be targeted to reduce mitochondrial stress.

## 1. Introduction

The mechanistic Target of Rapamycin (mTOR) is a central regulator of nutrient metabolism and glucose homeostasis. The mTOR protein is a highly conserved serine/threonine kinase that integrates extracellular and intracellular inputs from nutrients, insulin, growth factors, and environmental cues. In addition, it transmits signals to downstream targets and networks with multiple signaling pathways to control cell growth, survival, and metabolism [1,2].

mTOR has also been identified as a driver of stem cell growth and pancreatic progenitor cell differentiation [3,4]. The mTOR protein nucleates two functionally distinct and mutually exclusive complexes to regulate cell growth, energy metabolism, and survival, namely mTOR Complex 1 (mTORC1) and mTOR Complex 2 (mTORC2). mTORC1 binds specifically to Raptor and other protein partners, is a central hub for nutrient signaling, energy, and growth factors, and coordinates the anabolic cell growth and catabolic autophagy [1]. On the other hand, mTORC2 binds exclusively to Rictor and other proteins, which drives insulin signaling by activating Akt (Ser473) phosphorylation downstream of the PI3 kinase/insulin pathway [5]. In this manner, the mTOR network serves as a nutrient sensor and orchestrates energy metabolism. Moreover, the mTORC1 and mTORC2 complexes are dysregulated in several chronic diseases, including type 2 diabetes, insulin resistance, obesity, metabolic syndrome, and certain cancers [6]. As such, mTOR complexes and their downstream targets are actionable proteins and metabolic targets due to their integral role in energy metabolism and in pancreatic progenitor cell growth.

Specifically, mTOR complexes play a significant role in amino acid metabolism [7,8]. mTORC1 regulates amino acid metabolism via several amino acid sensors [8,9,10,11]. In addition, glutamine signaling is relayed via the Arf-1 rag-independent mechanism and drives the glutaminolysis pathway [12]. On the other hand, methionine is sensed via SAMTOR (S-adenosylmethionine sensor upstream of mTORC1). However, aspartate, asparagine, and glycine amino acid sensors have not been identified. Recently, Xu et al. suggested that asparagine may stimulate mTORC1 in brown adipose tissue [13]. Therefore, we applied the non-targeted metabolomics approach as a readout of the internal exposome and measured the levels of amino acids to gain insight into systems biology and mTORC1/mTORC2 mediated metabolic pathways enrichment.

**Figure 1:**
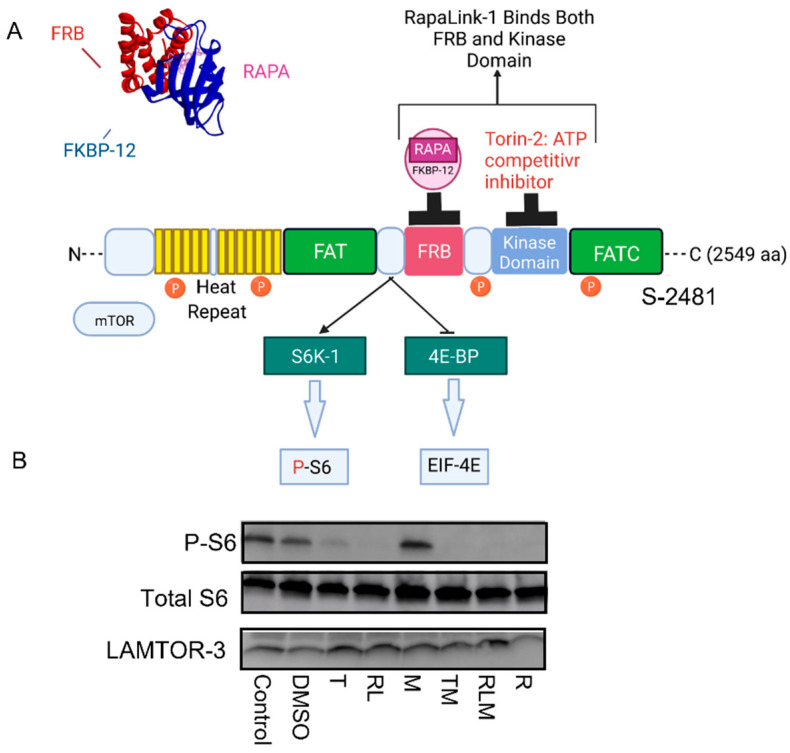
mTOR Signaling and mechanism of actions of mTOR inhibitors. (**A**) Binding of mTOR inhibitors to different domains on the mTOR protein. Rapamycin binds to the FKBP Rapamycin Binding (FRB) domain, and FKBP-12 forms a ternary complex that blocks mTORC1 functions; Torin-2 binds to the kinase domain, RapaLink-1 binds to both the FRB and the kinase domains. (**B**) RapaLink-1, Torin-2, rapamycin, and the combination of Torin-2 and metformin (TM) and RapaLink-1 and metformin (RLM) decreased mTOR signaling to downstream target P-S6.

Given the complex interactions between the mTOR network, amino acids, and nucleotide metabolic pathways, we hypothesized that mTORC1 and mTORC2 complexes coordinate the amino acid metabolites output and pathway enrichments in pancreatic islet β-cells. To test this hypothesis, we used pancreatic β-cell culture (Beta TC-6) to test the effects of a third-generation mTOR inhibitor, RapaLink-1 [14]—which bivalently links rapamycin with an mTOR kinase inhibitor (MLN0128) [15] and blocks both mTORC1 and mTORC2 (Figure 1); Torin-2, a second-generation mTOR competitive inhibitor of ATP; compared to rapamycin (R), the prototype mTOR inhibitor; and metformin, the indirect mTOR inhibitor (AMPK activator)—on the untargeted metabolomics as a measurement of the internal exposome, amino acid metabolites, and mitochondrial functions compared to the control. Furthermore, since rapamycin binds to the FKBP Rapamycin-Binding (FRB) on mTOR forming a ternary complex with FKBP-12, which inhibits only mTORC1, at least in the short-term, this approach allowed us to compare the effects of mTORC1 inhibition versus mTORC1 and mTORC2 and provide mechanistic insight into amino acid regulation by mTORC1 and mTORC2 (Figure 1).

In this study, we used β-TC-6 cells, which secret insulin in response to glucose [16], to investigate the differential effects of mTORC1/mTORC2 inhibitors on the untargeted metabolomics, amino acids levels, and mitochondrial bioenergetics.

## 2. Methods

### 2.1. Reagents

Reagents were obtained from the following sources: Torin 2 (9-(6-aminopyridine-3-yl)-1-(3-trifluromethyl)-phenyl) benzos [h] [1,6] naphthyridin-2 (1 H) (cat # 4248) was obtained from Tocris Bioscience (R & D Systems, Minneapolis, MN, USA). RapaLink-1 was purchased from MCE MedChem Express, Monmouth Junction, NJ, USA, Cat No.: HY-111373. Rapamycin was obtained from Cell Signaling (cat #9904). Metformin hydrochloride (*N*, *N*-dimethyllimidodicarbonimidic diamide hydrochloride) (Tocris, cat #2864) and other chemicals were obtained either from Sigma (St. Louis, MO, USA) or Thermo Fisher Scientific (Waltham, MA, USA). Immobilon-P polyvinylidene difluoride membrane (0.45 µm) and the reagents for enhanced chemiluminescence (ECL) were obtained from Millipore (Burlington, MA, USA) (Immobilon Western chemiluminescent horseradish peroxidase). High-performance liquid chromatography (HPLC)-grade methanol, acetonitrile, ammonium acetate, acetic acid, propylene glycol, and phosphate-buffered saline (PBS) (1×) were obtained from Fisher Scientific (Fair Lawn, NJ, USA) as previously described [17]. Isoflurane was obtained from Halocarbon Product Corporation (River Edge, NJ, USA).

### 2.2. Antibodies

Antibodies against the following proteins were purchased from Cell Signaling: total Akt (cat #4691); total mTOR (cat #2983); serine P-2481 mTOR (cat #2976); S6 (cat #2217); serine 235/236 phospho-S6 ribosomal protein (cat #2211). Sheep anti-rabbit secondary antibodies were obtained from G. E. Health Care Bioscience Corp. (Piscataway, NJ, USA).

### 2.3. Cell Lysis for Western Blotting Analysis

We used insulin-secreting, glucose-responsive, pancreatic beta cells derived from transgenic mice expressing SV40 (Beta-TC-6 cells, ATCC-CRL-11506). Pancreatic islet β cells (β-TC-6) were harvested in RIPA buffer, snap-frozen in liquid nitrogen, and stored at −80° degrees until ready for analysis. Cells were washed twice with ice-cold PBS (pH 7.4) and collected in ice-cold lysis buffer containing KPO_4_, 1 mM EDTA, 5 mM EGTA, 10 mM MgCl_2_, 50 mM β-glycerophosphate, 1 mM sodium orthovanadate (Na_3_VO_4_), 5 µg/mL pepstatin A, 10 µg/mL leupeptin, and 40 µg/mL phenylmethylsulfonyl fluoride (PMSF). Cells were sonicated for 30 s at 50% power using Branson Digital Sonifier 250 (Branson Ultrasonic, Danburg, CT) and stored at −20 °C until ready for use. Cell lysates were centrifuged at 13,200 rpm for 15 min at 4 °C, and the supernatants were collected. Protein concentration was measured with a Bradford assay. Immunoblotting was conducted as we described previously [2,18,19]. Briefly, samples were heated at 95 °C for 5 min and electrophoresed on SDS-PAGE gels to resolve the protein bands according to their molecular weight. The protein bands were transferred to polyvinylidene difluoride membranes in Tobin buffer (24 mM Tris, 192 mM glycine, 20% methanol). Western blotting was performed by blocking the membranes in TBST (40 mM Tris HCL, (pH 7.5), 0.9% NaCl, 0.1% Tween 20) containing 5% nonfat milk. The membranes were incubated in TBST with 5% nonfat milk containing the primary antibody and washed 3 times with TBST, followed by the addition of the secondary horseradish peroxidase-conjugated antibodies. The blots were developed via enhanced chemiluminescence (ECL, Signal Fire, Cell Signaling, CST) as previously described [2] and visualized using AI 600 Chemiluminescent imager.

### 2.4. Metabolites Extraction for the HRMS Untargeted Metabolomics Study

Pancreatic islet β cells (β-TC-6; CRL-11506) were plated in 60 mm dishes (1 × 10^6^ cells per plate) in complete DMEM media. The media was removed the following day, and fresh buffer was added equally to all plates. β-TC-6 cells (4 plates per treatment) were randomly assigned to (1) Control; (2) Torin-2 (100 nM, T); (3) RapaLink-1 (10 nM, RL); (4) M: metformin (1 mM, M); (5) Rapamycin (10 nM, R) for 24 h. The β-TC-6 cells were gently rinsed with diH_2_O and snap frozen in liquid nitrogen to quench the metabolism using a standard protocol for cell harvest [20]. Cells were extracted in ice-cold 50% ethanol and 50% diH_2_O solution, collected in microfuge tubes, and vortexed for 4 min at 13,000 rpm. The supernatant was then extracted in 90% ethanol and 10% water, vortexed, and stored at −20 °C until ready to use. The high-resolution untargeted metabolomics (HRM) was performed using LC-MS/MS HILIC peak detection of ESI positive and negative polarity modes.

### 2.5. LC/MS/MS High-Resolution Mass Spectrometry (HRMS) Untargeted Metabolomics

The data were collected using Bruker’s maXis-II ESI-Q-q-TOF coupled to Dionex Ultimate-3000 U(H)PLC system. This platform combines TOF technology with unique software packages that deliver sub-ppm mass accuracy (<1 ppm) and up to 80,000 isotopic mass resolution. The application includes LC-MS/MS (with CID and ETD capabilities). The analytical conditions used are SeQuant ZIC-HILIC 150 × 2.1 mm column (Bruker, Hamburg, Germany). Solvent A included 97% acetonitrile and 3% water with 7 mM ammonium acetate. Solvent B was 97% water and 3% acetonitrile with 7 mM ammonium acetate. The total gradient time was 30 min.

We applied the XCMS online cloud-based bioinformatics platform for mass spectrometry processing developed by the Scripps Institute to link mTOR-regulated metabolites in pancreatic islet β-cells data to the neural networks’ biological pathways using the mummichog algorithm [21,22,23,24]. The chromatogram parameters were applied using the XCMS online version 2.3.1 and camera version 1.30.1. The Centwave, Positive polarity was used for feature detection. The parameters included 10 ppm accuracy, and the obiwrap method was used for retention time. Grouping and alignments were conducted using the density method. After filling the Peaks, a differential report was generated to compare spectral differences between multiple groups using the ANOVA-on-Ranks statistical test (Kruskal Wallis, with a *p*-value threshold of 0.001), and a secondary post-hoc analysis was performed comparing every two groups. For system biology analysis, the Mummichog algorithm analysis of metabolic pathways was utilized [25]. Quality control was determined using an MDS plot and static PCA, and scaling plots were drawn. For annotation, both isotopes and adducts were analyzed using the following parameters, 5 ppm, sigma 6, maximum charge 3, and maximum isotope 4. Metabolites were identified based on accurate mass to charge unique *m*/*z* ratio measurements and matching their MS/MS fragmentation spectra with the available metabolite databases (METLIN standard database matching and MoNA).

### 2.6. Live Cells Bioenergetics

We used pancreatic islet β cells (β-TC-6; CRL-11506) and employed the third-generation mTOR inhibitor, RAPA-Link, (Rapamycin linked to mTOR ATP-competitive inhibitor) [26], Torin 2 (ATP competitive inhibitor of mTORC1 and mTORC2), rapamycin (the prototype mTOR inhibitor, mainly mTORC1 inhibitor via FRB domain inhibition), and the antidiabetic drug, metformin, which activates AMPK and indirectly blocks mTORC1, to determine the functional role of mTORC1 and mTORC2 networks on energy homeostasis and mitochondrial functions. The β-TC-6 cells (30,000 cells per well) were incubated with one of the following treatments for 24 h: (1) no treatment control, (2) DMSO control, (3) Torin-2 (100 nM, T), (4) RapaLink-1 (10 nM, RL), (5) metformin (1 mM, M), (6) Torin-2 (100 nM) + metformin (1 mM, TM), (7) RapaLink (10 nM) + metformin (1 mM, RLM), (8) Rapamycin (10 nM, R).

### 2.7. Live Cells Mitochondrial Functions

We measured the mitochondrial functions using Agilent seahorse XFe24 Live-Cell metabolism analysis (Agilent/seahorse XFe24 Analyzer). The live-cell bioenergetics was conducted to determine the basal mitochondrial functions, oxygen consumption rates (OCR), extracellular acidification rates (ECAR), ATP production, proton leak, maximal respiration, spare respiratory capacity, mitochondrial stress, and nonmitochondrial respiration using the standard manufacturers’ protocols [2,27,28].

### 2.8. Data Processing and Statistical Analysis

For the metabolomics study, the data were analyzed using the XCMS online cloud-based bioinformatics platform. The raw metabolomics spectra were uploaded and processed for peak detection, retention time, corrected chromatogram, alignments, and metabolite feature annotation. These features allow for linking the metabolomics data to metabolic pathways and biological systems [29,30,31,32].

We performed ANOVA on Ranks (Kruskal Wallis test) using SPSS software for parametric data to determine whether the treatment groups differed with respect to a given outcome compared with the control group. If the overall ANOVA and Kruskal Wallis tests were significant, a secondary post-hoc Welch *t*-test was employed to identify the pairs of groups that differed. The statistical significance was preset to *p* < 0.05.

The mitochondrial functions data were analyzed and expressed as the mean ± standard error (S.E.). For the parametric data, we applied analysis of variance (ANOVA) to determine the overall significance between groups, followed by an unpaired Welch *t*-test, unequal variance.

## 3. Results

### 3.1. mTOR Complexes Module Cell Signaling

Pancreatic islet β cells (Beta TC-6) were incubated for 24 h with different classes of mTOR inhibitors, as shown in Figure 1A. We used Western blotting analysis to show that metformin treatment alone (M) did not affect mTOR signaling to phospho ribosomal protein S6 (Figure 1B). However, Torin 2 (T), RapaLink-1 (RL), Rapamycin (R), and the combination of Torin-2 and metformin (TM) or RapaLink-1 and metformin (RLM) inhibited phosphorylation of the mTORC1 downstream target ribosomal protein S6 (Figure 1B). The total S6 and LAMTOR3 (Late Endosomal/Lysosomal Adaptor, MAPK, and mTOR Activator 3) protein expressions localized to the late endosome were similar between groups.

### 3.2. mTORC1 and mTORC2 and Amino Acid Levels

In this study, we measured all of the 20 biologically natural amino acids found in eukaryotes (Table 1) as part of the metabolome, including the nine essential amino acids that cannot be synthesized in the human body and must be consumed. Out of the 20 amino acids, we found 4 amino acid levels were statistically significantly different between groups as determined by ANOVA and ANOVA-on-Ranks.

**Figure 2:**
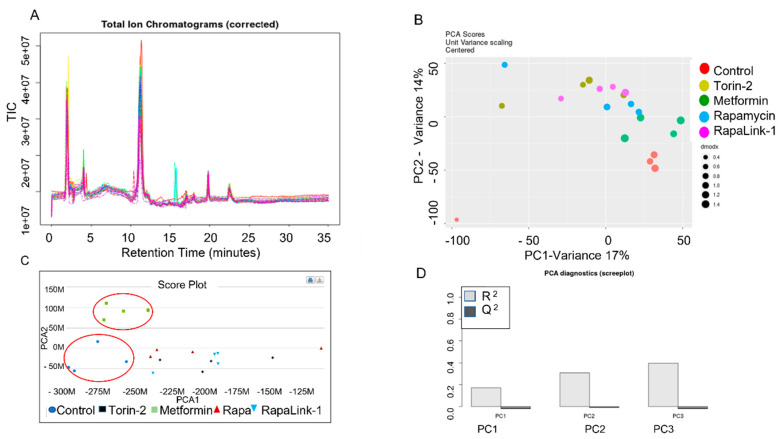
LC-MS/MS HILIC Total Ion Chromatograph (TIL), Alignment and Validation. (**A**) Total Ion Chromatogram (corrected), (**B**) PCA dimension reduction, and (**C**) Score Plot comparison between treatment groups. (**D**) PCA Diagnostics Data (Scree Plot).

These differences included L-aspartic acid (*p* = 0.009), L-asparagine (*p* = 0.04), L-glutamate (*p* = 0.01), and glycine (*p* = 0.03). Additionally, we applied the python algorithm and METLIN database to link the raw metabolomics data to functional interpretation enrichments of the metabolic pathways and integrate biological networks in pancreatic islet β cells. The initial preprocessing of ZIC-HILIC LC/MS data included MS peak detection, analysis of MS/MS data, retention time alignment, normalization, imputation, batch correction, and quality control (Figure 2A–D). First, we performed an exploratory multivariate analysis with unsupervised Principal Component Analysis (PCA) for dimension reduction and outlier identification. PCA dimension reductions show the clustering of each treatment group. Then, we visualized the dimensionality of the data by the scree plot. Finally, the Total Ion Chromatograms (TIC) were aligned, and PCA scores were centered and validated.

**Figure 3:**
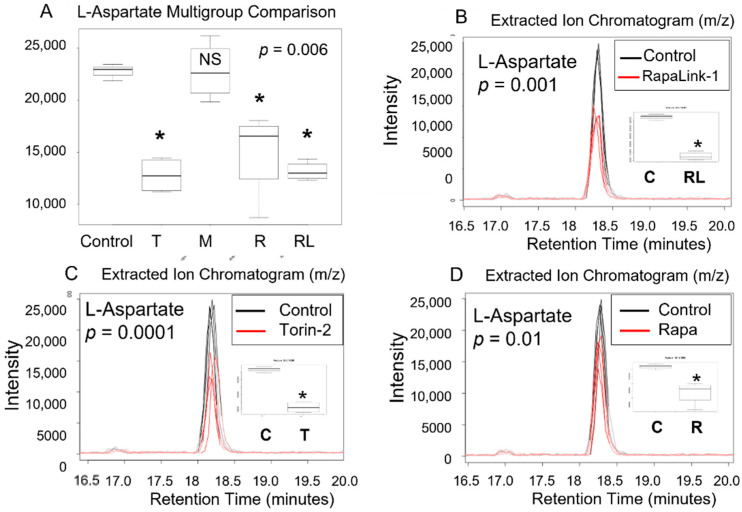
L -aspartic acid level decreased in pancreatic beta cells treated with mTORC1/mTORC2 inhibitors compared to > the control. Normal phase chromatography LC-MS/MS (HILIC) was used to compare groups. We conducted a multi-group comparison to identify groups’ differentially expressed metabolite features (**A**). (**B**) shows the differences of the intensity of the extracted ion chromatogram between Rapa-Link-1 and the control. (**C**) shows the differences in intensity between Torin-2 and the control. (**D**) Shows the differences between Rapamycin and the control. * *p* < 0.05. C, control; T, torin-2; M, metformin, R, rapamycin, RL, rapalink-1; NS, no significance.

**Figure 4:**
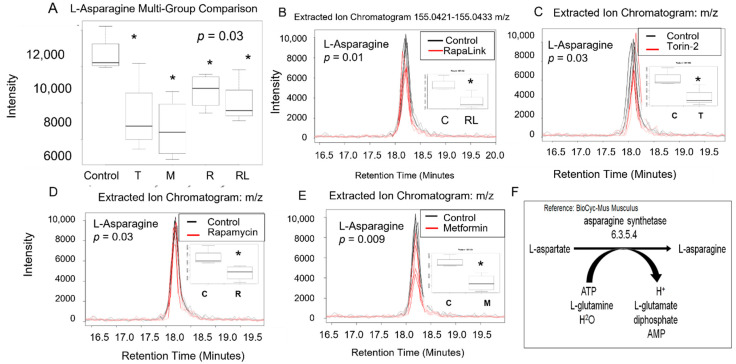
The L-asparagine level decreased in pancreatic beta cells treated with mTORC1/mTORC2 inhibitors compared to the control. Normal phase chromatography LC-MS/MS (HILIC) was used to measure the level of L-aspartic acid. (**A**) shows the multigroup comparison in the Extracted Ion Chromatogram Intensities. The difference in the extracted ion chromatogram intensity between RapaLink-1 treatment and the control was shown in (**B**), the level change induced by Torin-2 treatment was shown in (**C**), and the level change induced by rapamycin was shown in panel (**D**). Changes induced by metformin were shown in panel (**E**). Multigroup comparison by non-parametric ANOVA on ranks test was conducted, followed by post- hoc Welch unpaired *t*-test. * *p* < 0.05. (**F**) shows the biochemical pathway of the conversion of L-aspartate to L-asparagine. C, control; T, torin-2; M, metformin, R, rapamycin, RL, rapalink-1.

We conducted a multigroup comparison to identify the differentially expressed metabolite features across all groups (Table 1), followed by a post-hoc analysis of each two groups (Figure 3 and Figure 4).

Our data in pancreatic beta TC-6 cells revealed that treatment with mTOR inhibitors significantly decreased amino acid L-aspartate level compared to the control (*p* = 0.006, ANOVA on Ranks (Kruskal Wallis test), Table 1, Figure 3A). Furthermore, using an unpaired parametric Welch *t*-test post-hoc test, we show that RapaLink-1 significantly decreased L-aspartate (*p* = 0.001), Torin-2 (*p* = 0.0001), and rapamycin (*p* = 0.01) compared to the control. On the other hand, metformin alone did not show any statistical difference compared to the control (Figure 3B–D).

All mTOR inhibitors, as well as metformin, decreased levels of L-asparagine compared to the control. Since L-aspartate is converted to L-asparagine in a reaction catalyzed by asparagine synthetase enzyme in the presence of ATP, L-glutamine, and H_2_O to form L-asparagine and L-glutamate (Figure 4), we investigated the impact of mTOR inhibitors on Beta TC-6 pancreatic cells on L-asparagine and L-glutamate levels (Figure 3 and Figure 4).

The multigroup comparison showed that incubating pancreatic beta TC-6 cells with mTOR inhibitors led to statistically significant differences in L-asparagine levels compared to the control (*p* = 0.03, ANOVA on Rank, Kruskal Wallis test, Table 1, Figure 4A). Using the post-hoc Welch unpaired *t*-test, we showed that compared to the control, RapaLink-1 significantly decreased L-aspartate (*p* = 0.01), Torin-2 (*p* = 0.03), rapamycin (*p* = 0.03), and metformin (*p* = 0.009) compared to the control (Figure 4B–E). We did not find differences in L-glutamine levels between groups (Table 1), indicating that L-aspartate and not L-glutamine is the primary driver of L-asparagine and L-glutamate formation. These data also suggest that L-asparagine is regulated by more than one mechanism, including mTOR and AMP pathways. The predictive pathway that is affected is represented in Figure 4F.

**Figure 5:**
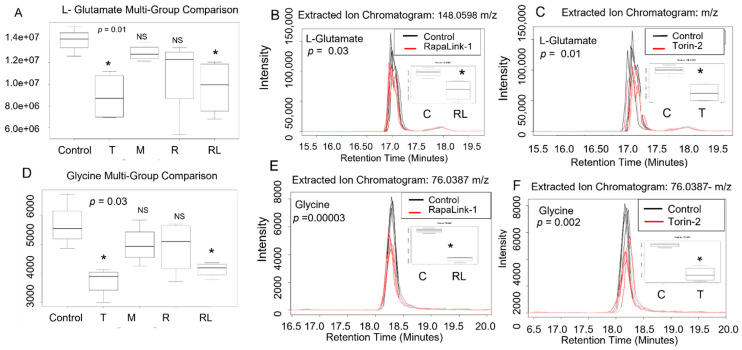
L-glutamate and glycine levels decreased in pancreatic beta cells treated with mTORC1/mTORC2 inhibitors compared to the control. Normal phase chromatography LC-MS/MS (HILIC) was used to compare groups. We conducted a multi-group comparison to identify groups’ differentially expressed metabolite features. (**A**) shows multigroup comparison in L-glutamate chromatogram intensity. (**B**) shows the differ-ences in intensity between RapaLink-1 and the control. (**C**) Shows the differences between Torin-2 and the control. (**D**) shows multigroup comparison in L-glycine chromatogram intensity. (**E**) shows the differences in intensity between RapaLink-1 and the control. (**F**) Shows the differences between Torin-2 and the control. * *p* < 0.05. C, control; T, torin-2; M, metformin, R, rapamycin, RL, rapalink-1; NS, no significance.

There was an overall significant difference in L-glutamate (*p* = 0.01) and L-glycine (*p* = 0.005) levels between the treatment groups and the control (*p* = 0.01) (Table 1). Using the post-hoc unpaired parametric Welch *t*-test, RapaLink-1 significantly decreased L-glutamate levels (*p* = 0.03) and Torin-2 (*p* = 0.01) compared to the control. In contrast, rapamycin and metformin did not alter L-glutamate levels (Figure 5A,B). Similarly, beta TC-6 cells incubated with mTOR inhibitors data showed an overall difference in L-glycine levels between the treatment groups and the control (*p* = 0.005) (Table 1). Using the post-hoc Welch *t*-test, RapaLink-1 significantly decreased L-glycine levels (*p* = 0.00003) and Torin-2 (*p* = 0.002) compared to the control. Rapamycin and metformin did not alter L-glycine levels in the same manner as the data related to L-glutamate levels (Figure 5C,D).

### 3.3. Dysregulated Metabolic Pathways and the Metabolic Activity Network Predictive Model

**Figure 6:**
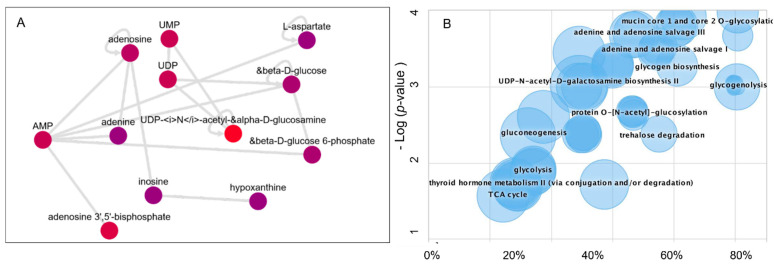
Metabolomics data processing using the Systems Biology platform. (**A**) Metabolic Activity Network model (**B**) The Generated Dysregulated Pathways cloud plot.

The raw LCMS/MS data were processed using the XCMS biological systems module, which applies the mummichog algorithm to identify the metabolic activity network and dysregulated pathways. Thus, this system enables the biological interpretations of the untargeted metabolomics data (Figure 6). The metabolites information was queried using BioCyc integration with the METLIN database. The predicted metabolic activity network in Beta-TC6 in response to mTORC1/mTORC2 inhibition is shown in Figure 6A. In addition, the pathway cloud plot was generated to visualize the dysregulated metabolic pathways (Figure 6B) based on the differential expression of amino acids L-aspartate, L-asparagine, L-glutamate, and glycine with mTORC1/mTORC2 inhibition (Figure 3, Figure 4 and Figure 5). The cloud-plot data analysis for identifying differentially expressed metabolites showed that mTORC1 and mTORC2 inhibition altered various metabolic pathways, including protein O-N acetyl glycosylation, adenine, and adenosine salve pathway, gluconeogenesis, glycogenolysis, and glycogen biogenesis.

Our data support the notion that L-aspartate and AMP interact to regulate adenine, adenosine nucleotides, hypoxanthine, UDP-glucosamine, UDP, glucose metabolism, and ATP-activated glucose-6-phosphate.

### 3.4. Live-Cell Bioenergetics

**Figure 7:**
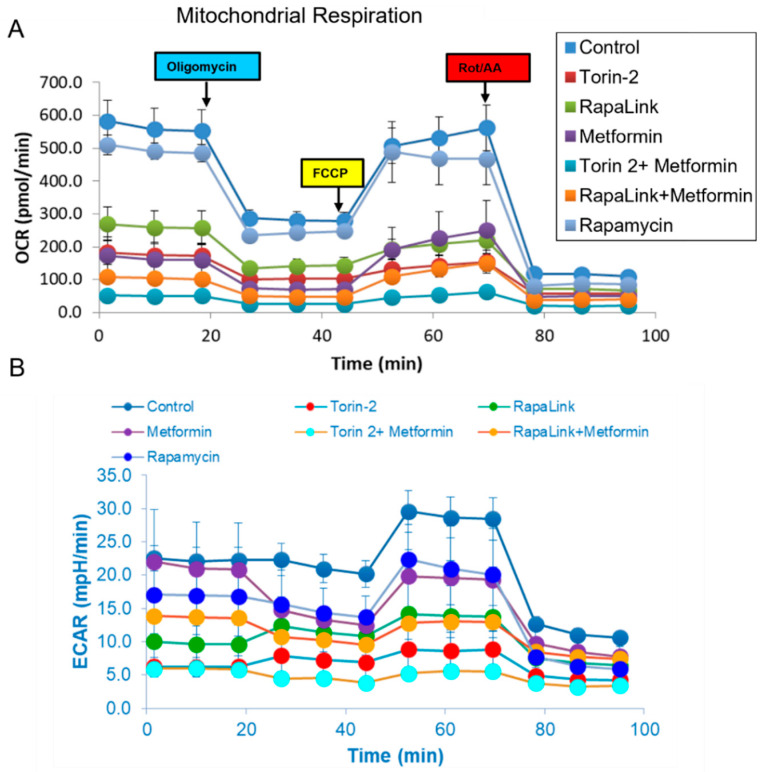
Mitochondrial stress test. Oxygen consumption rate (OCR) and extracellular acetylation rates (ECAR) comparison between groups. (**A**) Comparison of the live-cell oxygen consumption rate (OCR) indicates the mitochondrial oxidative phosphorylation and (**B**) Extracellular esterification rate (ECAR) reflects the anaerobic glycolysis in Beta-TC6 bioenergetics analysis incubated with different treatment groups.

**Figure 8:**
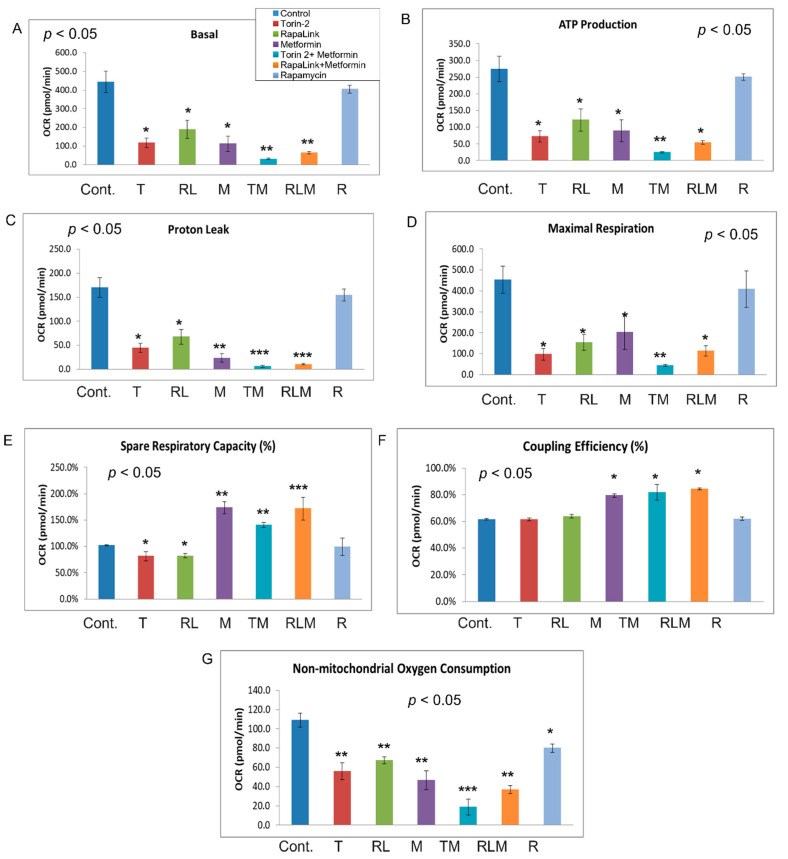
Mitochondrial stress test. Bioenergetic dynamics and cellular metabolism were quantified using MitoStress test XFe24 (Agilent, Seahorse). The real-time, live-cell approach simultaneously measures oxygen consumption rate (OCR) and extracellular acidification rate (ECAR) to determine cellular respiration and metabolism, including basal respiration (**A**), ATP production (**B**), proton leak (**C**), maximal respiration (**D**), spared respiratory capacity (**E**), coupling efficiency (**F**), and non-mitochondrial oxygen consumption (**G**). ANOVA was applied to determine the statistically significant differences between each treatment and the control, followed by an ad-hoc secondary unpaired t-test. * *p* < 0.05, ** *p* < 0.01, *** *p* < 0.001.

To address the mechanistic underpinning of these results, we conducted live-cell mitochondrial functional studies, as shown in Figure 7. In the activity network predictive model and pathway analysis, we conducted Beta-TC6 live-cell bioenergetics studies to determine the differences in oxygen consumption rates (OCR), which are indicative of mitochondrial oxidative phosphorylation, and extracellular acidification rate (ECAR), which is suggestive of nonmitochondrial anaerobic glycolysis (Figure 7A,B). With the addition of oligomycin, which inhibits ATP synthase (Complex V) in the mitochondrial Electron Transport Chain, the impact did not differ between cells treated with rapamycin and the control (Figure 7 and Figure 8).

In comparison, all other treatment groups showed a difference in ATP production (Figure 7A and Figure 8B). Furthermore, the FCCP, which uncouples the oxidative phosphorylation by disrupting the mitochondrial membrane potential and collapsing the proton gradient, had a similar effect in rapamycin-treated cells and the control (Figure 7 and Figure 8). On the other hand, injection of a mixture of rotenone (electron transport chain complex I inhibitor), and Antimycin A (Complex III inhibitor), which shuts down mitochondrial respiration and therefore enables the calculation of nonmitochondrial respiration, showed a significant difference between the control and all other groups including rapamycin-treated cells (Figure 8G). The data further showed that Rapalink-1, Torin-2, and metformin altered mitochondrial functions in pancreatic islet beta cells by decreasing the basal respiration (Figure 8A), ATP production (Figure 8B), proton leak (Figure 8C), maximum respiration (Figure 8D), and spare respiratory capacity (Figure 8E).

On the contrary, rapamycin did not alter these mitochondrial function parameters compared to the control. Rapamycin only decreased the nonmitochondrial respiration compared to the control (Figure 8G), but still less than RapaLink-1, Torin-2, and metformin (Figure 8G). The coupling efficiency was slightly elevated with metformin and the combination of metformin and Torin-2 or RapaLink-1 but not with mTOR inhibitors alone (Figure 8F). As mentioned earlier, rapamycin had no significant effect on mitochondrial functions but decreased nonmitochondrial oxygen consumption (Figure 7 and Figure 8). The data showed that mTORC1/mTORC2 chemical knockout decreased ATP production, nonmitochondrial oxygen consumption, and proton leak.

## 4. Discussion

This study sought to identify the amino acid metabolites whose levels might be regulated by mTORC1 and mTORC1 complexes by employing the high-resolution untargeted metabolomics and live-mitochondrial functions in pancreatic beta cells. While the mTOR inhibitors’ immediate application is pharmacological drug use, the advantage of using mTORC1 and mTORC2 inhibitors as tools is gaining insight into the mechanistic pathways that orchestrate cell anabolism and catabolism, which in turn inform nutrient metabolism and allows for the development of strategies for chronic disease interventions. Therefore, we chemically knocked out the mTOR complexes with drugs to determine their functionality, monitor the mTORC1/mTORC2 signaling network’s inner workings, and advance disease therapeutics. As such, we compared the effects of RapaLink-1 (third generation mTORC1/mTORC2 inhibitor), Torin-2 (second generation mTORC1/mTORC2, ATP-competitive inhibitor), and the prototype mTOR inhibitor, rapamycin, which inhibits mTORC1 in most cell lines with few exceptions, metformin (AMPK activator), which indirectly inhibits mTOR to control groups. We linked the resultant untargeted metabolomics spectra and peak annotation with metabolic pathways and biological networks. We further leveraged the high-resolution liquid chromatography/mass spectrometry (LC-MS) to link aspartate metabolism and raw metabolomics spectra changes with network visualization and biological pathways [21,22,23,33,34,35,36,37,38]. As expected, mTOR inhibition decreased the downstream targets’ phosphorylation (Figure 1B).

Here we reported that mTORC1 and mTORC2 complexes regulate L-aspartate, L-asparagine, L-glutamate, and glycine levels compared to the control in pancreatic islet beta cells. We report that mTORC1 and mTORC2 complexes regulate L-aspartate, L-asparagine, L-glutamate, and glycine levels compared to the control in pancreatic islet beta cells (Figure 3, Figure 4 and Figure 5). Given that aspartic acid is the carbon source in purine synthesis and glycine and aspartate incorporation in pyrimidine, it is possible the mTOR regulates the incorporation of these amino acids in purine and pyrimidine bases and thus nucleotides metabolism. Further, since glycine and glutamate are required for glutathione synthesis, it is possible that mTOR complexes may play a role in the antioxidant capacity of glutathione. Our study leveraged high-resolution metabolomics to link mTOR-associated metabolomics raw spectra with metabolic pathways with predicted pathway enrichment biologic network visualization (Figure 6A,B).

As mentioned earlier, mTORC1 receives inputs from amino acids, amino acid transporters, and input from glucose via insulin signaling and responds to energy levels. As such, mTORC1 controls amino acid and protein synthesis and energy metabolism, which leads to the activation of glycolysis and the TCA cycle, and the generation of ATP. This energy is used to drive anabolic pathways and imped catabolic pathways. mTORC1 regulates amino acid metabolism via several amino acid sensors. For example, leucine is sensed via Sestrins sensors [10]. Similarly, CASTOR also senses arginine by disrupting CASTOR1 and CASTOR 2 via GTP-RagA and GDP-RagC heterodimerization [9]. Further, arginine is sensed via the SLC38A9 lysosomal transporter, which mediates the efflux of arginine from the lysosome [11]. In contrast, glutamine signaling is relayed via the Arf-1 rag-independent mechanism and drives the glutaminolysis pathway [12]. In addition, the sulfur-containing methionine is sensed via SAMTOR (S-adenosylmethionine sensor upstream of mTORC1), a GATOR1/KICSTOR-interacting protein. As such, S-adenosyl methionine binds to SAMTOR and interrupts SAMTOR-GATOR1 interaction, which is a negative regulator of mTORC1.

Despite the discovery of all these amino acid sensors, no aspartate or asparagine amino acid sensors have been identified to date. The gap in knowledge in amino acid metabolism stems from two possibilities: First, whether the sensor mentioned above is specific to one amino acid or can crosstalk and detect related amino acids. It is known that some amino acids are more potent in mTORC1 activation than others, so these amino acid sensors may be specific to individual amino acids. Second, it is unknown whether amino acid sensing is tissue-specific, whether mTORC1 senses all amino acids, or if there are mTORC1 independent pathways. For example, a recent study showed that asparagine might affect glycolysis in brown adipose tissue via mTORC1 activation [13]. In this study, we conducted a multigroup comparison followed by a post-hoc secondary analysis to determine the differential effects of different treatment group modalities that altered amino acid metabolism. We report that mTORC1 and mTORC2 regulate L-aspartate, L-asparagine, L-glutamate, and L-glycine levels. This novel observation warrants further research to understand basic physiology and biology and identify the scientific bases for disease management.

To gain insight into the mTOR-regulated bioenergetics, we used seahorse live-cell imaging to determine the impact of mTORC1/mTORC2 inhibition on mitochondrial functions. This system enabled us to determine the differences in oxygen consumption rates (OCR), indicative of mitochondrial oxidative phosphorylation and extracellular acidification rate (ECAR), which suggest anaerobic glycolysis. In real-time, this system allowed us to characterize and analyze cell metabolism phenotype, mitochondrial respiration and glycolysis, oxygen consumption rates (OCR), and extracellular acidification rate (ECAR). The addition of oligomycin, which inhibits ATP synthase (Complex V) in the mitochondrial Electron Transport Chain, did not affect cells treated with Rapamycin (Figure 6A and Figure 7B). In comparison, all other treatment groups showed a difference in ATP production (Figure 6A and Figure 7B). These findings suggest that rapamycin inhibition of the FRB domain does not alter mTOR catalytic activity. Despite the observation that both RapaLink and Rapamycin treatment lead to decreased aspartate and asparagine levels, the two mTOR inhibitors had different bioenergetics profiles (Figure 8A–E). While RapaLink decreased basal oxygen consumption (Figure 8A), ATP production (Figure 8B), proton leak (Figure 8C), maximal respiration (Figure 8D), and spare respiratory capacity (Figure 8E). These observations could be explained by various mTOR inhibitors’ different modes of action. Rapamycin only binds to the FRB domain, making a ternary complex with FKBP12 as FRBP-12-Rapamycin-FRB complex, which does not alter the mitochondrial functions.

Several papers reported that rapamycin decreased mitochondrial capacity in Jurkat cell lines [39] and primary human trophoblasts [40]. However, our study found that rapamycin decreased OCR oxygen consumption rate by reducing nonmitochondrial oxygen consumption, not through ATP production (Figure 7). This finding is consistent with the mechanism of action of rapamycin which functions by blocking the FRB domain rather than inhibiting the mTOR kinase (PI3Kinase catalytic domain) as shown in Figure 1 mechanism of action, while Rapalink-1 which blocks both the catalytic domain and FRB domain lead to a significant reduction in mitochondrial functions. It is also possible that rapamycin effects are cell-specific and time-specific. Indeed, investigators have documented biphasic response to TSC ablation upstream of mTOR in pancreatic beta cells [41]. Moreover, a study utilizing dynamic modeling of mTOR signaling revealed the biphasic dependence of mTORC1 on mTORC2 [42]. Rapamycin has also been shown to have a biphasic effect on insulin sensitivity in C2C12 myotubules [43].

On the other hand, RapaLink-1 binds to both FRB domains and the mTOR kinase domain as MLN0128, which explains the effects of Rapalink-1 on mitochondrial functions. Similarly, Torin-2, which competitively inhibits the mTOR kinase domain, has similar inhibitory effects on mitochondrial functions (Figure 8A–E). Thus, the data provided mechanistic information about the mTOR complexes’ role in small molecular metabolites, localization, and mitochondrial functions. In addition, the data showed that mTOR inhibition decreased ATP production, nonmitochondrial oxygen consumption, and proton leak.

Our findings provided a mechanistic underpinning and biological insight to show how mTORC1 and combined mTORC1 and mTORC2 chemical knockout alter the metabolomics phenotype and links to mechanistic pathways and biological networks. The data support the notion that the metabolomics profile can be applied for risk assessment, and early detection and metabolic laboratory tests can be utilized for precision nutrition and personalized medicine. mTORC1 and mTORC2 regulated metabolites could potentially be useful metabolic biomarkers as mTOR serves as a metabolic sensor and plays an integral role in cellular growth and metabolism.

## 5. Conclusions

Findings from these studies enabled us to determine the causal contribution of mTORC1 and combined mTORC1/mTORC2 to pancreatic islet β cells’ metabolic profile by combining integrative bioinformatics and laboratory approaches. Our data indicate that mTORC1/mTORC2 inhibition by RapaLink-1 and Torin-2 reduced L-aspartate and L-asparagine in pancreatic beta TC-6 cell lines. The mechanism of action is through both mTORC1 and mTORC2 inhibition of mitochondrial functions and decreasing ATP production. Rapamycin alone does not affect the basal mitochondrial function or ATP production in this pancreatic beta-cell model. Our findings provided a mechanistic underpinning and biological insight to show how mTORC1 and combined mTORC1 and mTORC2 inhibition regulate the metabolomics phenotype and link the mechanistic pathways with biological interpretations. The data support the notion that the metabolomics profile as a proxy of the internal exposome can be applied for risk assessment and early detection, and metabolic laboratory tests can be utilized for personalized medicine.

## Figures and Tables

**Table 1:** LC-MS/MS Multigroup Comparison between all the Natural 20 Amino Acids.

Amino Acid	Mass/Ion	Retention Time (RT)	Intensity					*p*-Value
	(m/z)		Control	Torin-2	Metformin	RAPA	RapaLink-1	
Glycine	98.02123	18.29						**0.034**
L-Alanine	90.05473	9.08						0.077
L-Serine	106.0497	18.34						0.279
L-Proline	116.0705	16.76						0.056
L-Valine	118.086	19.97						0.307
L-Threonine	120.0654	18.53						0.296
L-Cysteine	122.0268	16.83						0.154
L-Leucine	132.1018	13.51						0.676
L-Isoleucine	132.1019	3.31						0.056
L-Asparagine	133.0609	18.19						**0.042**
L-Aspartate	134.0448	17.01						**0.009**
L-Glutamine	147.0763	17.94						0.307
L-Lysine	147.1133	2.188						0.182
L-Glutamate	148.0604	17						**0.012**
L-Methionine	172.0402	14.32						0.22
L-Histidine	156.0766	19.62						0.731
L-Phenylalanine	166.0862	12.9						0.253
L-Arginine	175.1188	22.19						0.057
L-Tyrosine	182.0811	15.21						0.089
L-Tryptophan	205.0972	13.55						0.329

Notes: EIC is from the multigroup positive polarity comparison ID #1428608. Heatmap scale −2 (Blue), 0 (white) to +2 (Red). The bold font is the show the values were statistically significant.

## Data Availability

Not applicable.

## Abbreviations

mechanistic Target of Rapamycin Complex 1 (mTORC1); mechanistic Target of Rapamycin Complex 2 (mTORC2); High-Resolution Mass Spectrometry (HRMS); High Resolution Metabolomics (HRM); Mitochondrial stress (Mitostress); Oxygen Consumption Rates (OCR); Extra Cellular Acidification Rate (ECAR); Glycolysis; TCA (Tricarboxylic Acid) cycle; Metformin (M); RapaLink-1 (RL); Torin-2 (T); Rapamycin (RAPA); FKBP-12-Rapamycin-binding domain (FRB).
